# Grip Strength Estimation Using Input Data From a Commodity Smartphone: Model Development and Validation Study

**DOI:** 10.2196/90316

**Published:** 2026-04-23

**Authors:** Komei Tajima, Kaori Ikematsu, Toshiya Isomoto, Kunihiro Kato, Yuta Sugiura

**Affiliations:** 1 Department of Information and Computer Science Faculty of Science and Technology Keio University Yokohama, Kanagawa, Yokohama Japan; 2 LY Corporation Tokyo Japan; 3 Japan Women's University Tokyo Japan

**Keywords:** grip strength, mobile health, smartphone, machine learning, human computer interaction

## Abstract

**Background:**

Grip strength is a crucial indicator of muscle deterioration, recovery, sarcopenia, and neurological disorders. However, conventional measurement requires a dedicated dynamometer, which limits accessibility and requires specific movements.

**Objective:**

This study aimed to propose and validate a method for estimating grip strength using standard smartphone operations, thereby eliminating the need for specialized equipment.

**Methods:**

Data were collected from 21 young adults in the main experiment, who performed standard smartphone tasks (tapping, flicking, and dragging) after measuring their grip strength with a dynamometer. A predictive regression model was developed using touch and inertial sensor data. The model was first evaluated using a random split of the entire dataset (random split evaluation). To further assess practical feasibility and generalizability, we conducted leave-one-user-out validation and a few-day calibration validation; the latter simulated a calibration scenario by incorporating 1 to 4 days of user-specific data into the training set.

**Results:**

The regression analysis using a random split of the dataset demonstrated high accuracy, with a mean absolute error of 2.62 (SD 0.18) kg, a mean absolute percentage error (MAPE) of 8.91% (SD 0.57%), and a coefficient of determination of 0.802 (SD 0.036). In the validation of practical scenarios, the leave-one-user-out validation resulted in a MAPE of 15.08% (SD 5.40%). However, the personalized few-day calibration model showed significant improvements as calibration days increased, with the MAPE decreasing to 13.96% (SD 5.57%) after 1 day and reaching 11.64% (SD 5.80%) after 4 days. Furthermore, the National Aeronautics and Space Administration Task Load Index assessment indicated a low overall subjective workload (mean 3.04, SD 2.23 on a scale of 10), confirming the method’s suitability for daily use without a significant burden on users.

**Conclusions:**

The proposed method demonstrates that smartphones can serve as a viable, pervasive tool for daily grip strength monitoring, offering a convenient alternative to traditional dynamometers.

## Introduction

Muscle strength is known to decline with aging [1,2], and this decline is used as a diagnostic indicator of sarcopenia and frailty [3,4]. Among various muscle strength measures, grip strength is widely used in medical settings for treatment and rehabilitation [5-7]. Additionally, grip strength has been linked to chronic diseases, with several studies reporting a decline in grip strength among patients with diabetes [8-11]. Furthermore, reduced grip strength has been associated with an increased risk of falls and fractures, highlighting its relevance in assessing overall musculoskeletal health [12]. Therefore, regularly measuring grip strength and monitoring daily variations are essential for health management and disease risk assessment.

However, grip dynamometers are not widely available to the general public, and high-precision devices used in medical settings are expensive, making them difficult to obtain for personal use. Furthermore, electronic grip dynamometers require maintenance and are not easily portable, making measurements outside clinical settings challenging. These limitations hinder the feasibility of continuous and routine monitoring, potentially posing a barrier to effective health management.

**Figure 1:**
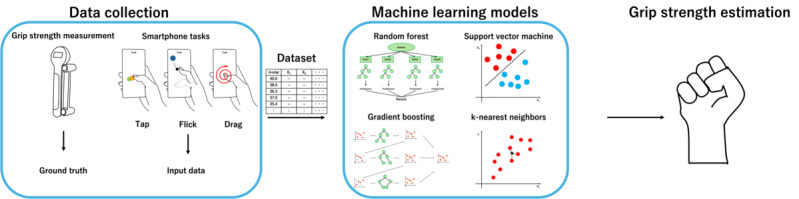
Overview of the proposed grip strength estimation method. Grip strength measurements and smartphone tasks were conducted, and the resulting data were used to construct a machine learning model. Grip strength values were set as the ground truth, while input coordinates, time, and sensor data were used as the input, enabling estimation through regression analysis.

We propose a method for estimating grip strength that leverages the widespread availability of commodity smartphones (Figure 1). Specifically, it uses simple and quick touch input tasks to provide practical and accessible grip strength measurements. For model construction, grip strength is first measured using a Jamar-style dynamometer to obtain the ground truth, followed by a smartphone task to collect the corresponding input data. This task involves common smartphone input gestures, such as tapping, flicking, and dragging, during which input coordinates, time stamps, contact area, and sensor data—including acceleration, angular velocity, and orientation—are recorded as feature variables. A regression model is then constructed using the measured grip strength as the target variable and the extracted features as explanatory variables. In total, 21 participants took part in the experiment, and the analysis results showed that the best-performing model had a mean absolute error (MAE) of 2.62 (SD 0.18) kg, a mean absolute percentage error (MAPE) of 8.91% (SD 0.57%), and a coefficient of determination of 0.802 (SD 0.036).

This method enables grip strength estimation using a regular smartphone without the need for specialized equipment, making daily measurements more accessible, enabling use in unsupervised environments, and requiring only a short amount of time (approximately 30 seconds per set of smartphone tasks). A brief overview of the study, including the experimental tasks and key results, is provided in Multimedia Appendix 1.

## Methods

### Recruitment

This study included 21 participants (n=16, 76.2% male and n=5, 23.8% female participants), aged 20 to 24 (mean 21.5, SD 1.00) years, with no functional impairments in their hands. All participants were right-handed. We provided participants with a prior explanation of the study and obtained their informed consent before participation.

### Procedure

**Figure 2:**
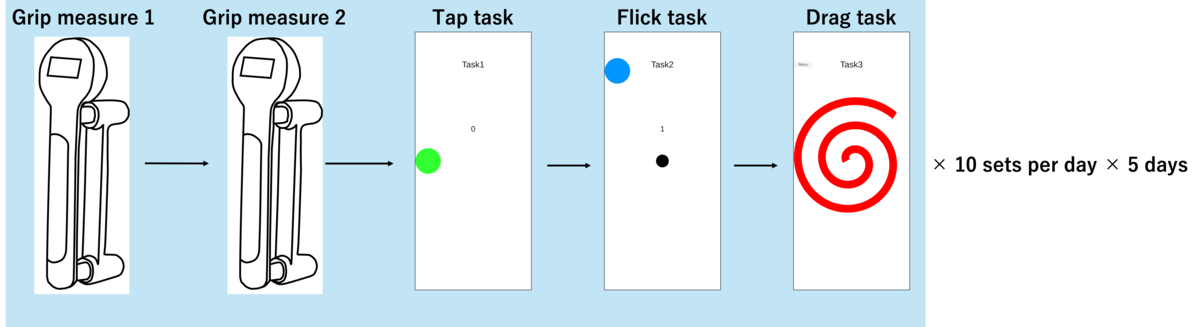
Overview of the data collection experiment. The experiment consisted of grip strength measurements and smartphone tasks (tap, flick, and drag).

Figure 2 shows an overview of the data collection experiment. Each day, participants performed a set of grip strength measurements and smartphone tasks (tap, flick, and drag tasks) 10 times. There were no breaks between the grip strength measurements and tasks, ensuring continuous execution; this design was deliberately implemented based on previous findings [13] that report a decrease in grip strength immediately after using a handgrip dynamometer, thereby intentionally inducing variability in grip strength over the 10 consecutive task sets. The measurements were conducted over 5 separate days, with at least a 2-day interval between each measurement day. The experimental procedures were standardized across all participants and measurement days.

### Grip Strength Measurement

**Figure 3:**
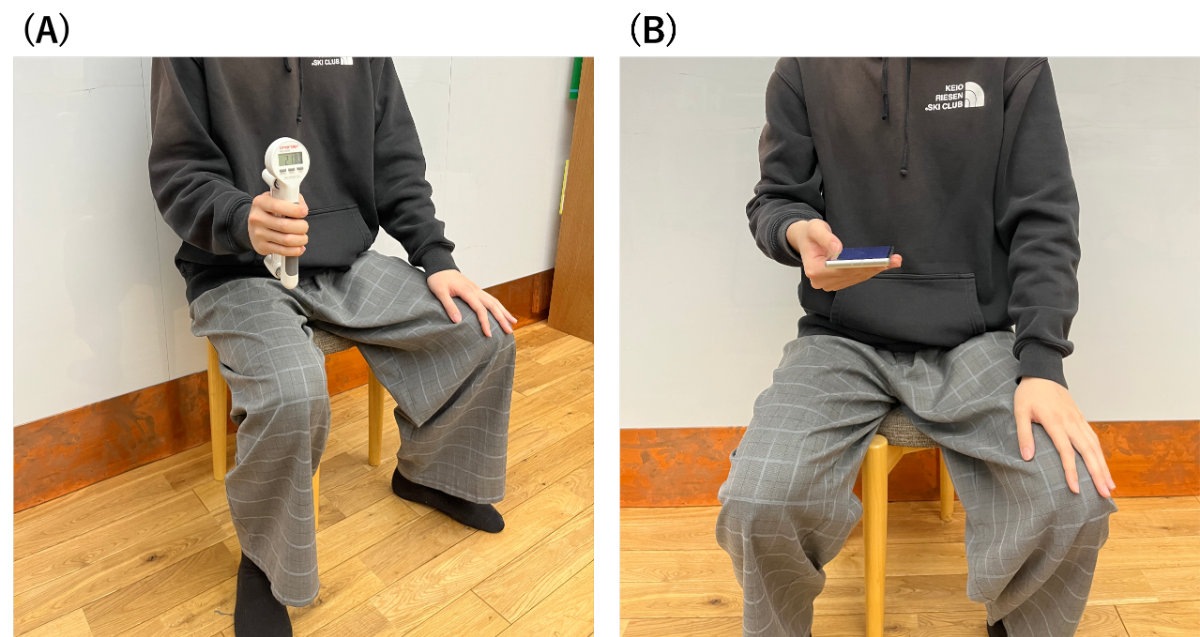
Postures used for (A) grip strength measurement and (B) smartphone tasks.

We used a Jamar-style dynamometer with a standard procedure to measure grip strength. Participants sat on a stable chair without armrests in a relaxed state during the measurement. During the measurement, they kept their upper limb on the measurement side aligned with their body and maintained a posture with the elbow joint flexed at 90° (Figure 3A). The measurement was conducted only on the dominant hand. After the first measurement, a second measurement was taken following a 10-second interval.

### Smartphone Task

**Figure 4:**
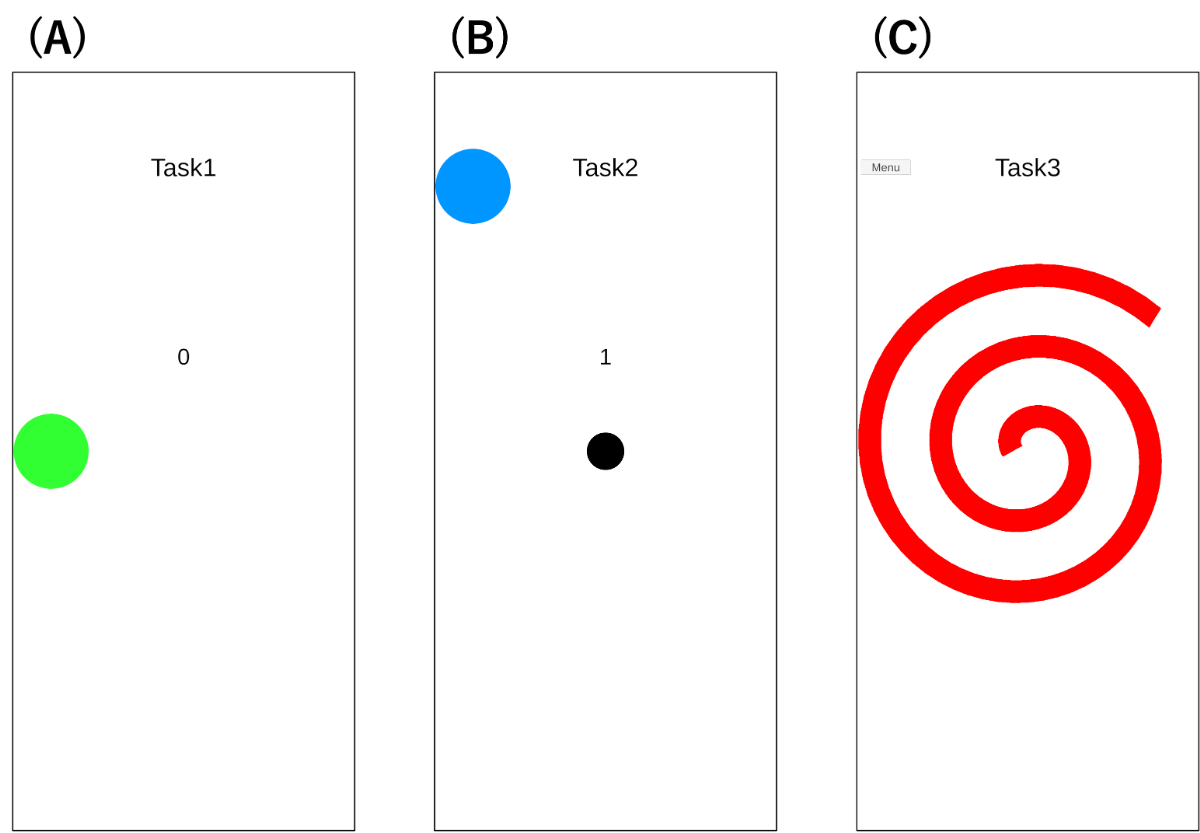
Screenshots of the experimental app used for data collection, illustrating (A) the tap task, (B) the flick task, and (C) the drag task.

A Google Pixel 7a (72.9 mm × 152.0 mm × 9.0 mm) was used for the task, and a custom-developed app built with Unity (version 2022.3.9f1; Unity Software Inc) was used. During the task, data from the device’s accelerometer, gyroscope, and orientation sensor were recorded at 60 Hz. Participants sat on a stable chair without a backrest, held the smartphone in their dominant hand, and operated it using their thumb. They were instructed to keep their hand elevated, ensuring that they did not touch the table or their lap (Figure 3B). Additionally, they were asked to perform the following three tasks with careful and deliberate movements: tap task, flick task, and drag task (Figure 4).

#### Tap Task

Participants tapped on a green target (14.6 mm diameter) displayed on the screen. When the target was tapped, a new target appeared at a different location. The task ended after 10 targets were tapped (Figure 4A).

#### Flick Task

Participants flicked a black circle (7.3 mm diameter) in a specified direction to make it collide with a blue target (14.6 mm diameter). When a successful collision occurred, a new target was generated. The task ended after 10 correct flicks in the designated direction (Figure 4B).

#### Drag Task

Participants traced a spiral (4.4 mm width) displayed on the screen from the center outward. When using the right hand, a clockwise spiral was used, whereas a counterclockwise spiral was used for the left hand. The task ended after tracing the spiral once (Figure 4C).

### Features

We extracted specific behavioral features from each task—tap, flick, and drag—to capture the kinematic characteristics associated with grip strength. For the tap task, the extracted features included the spatial accuracy, defined as the distance between the input coordinates and the center of the target, as well as temporal metrics, such as the time taken to eliminate the target and the duration of the touch interaction. The finger contact area during the tap was also recorded. In the flick task, we calculated the direction of the flick input (represented by x- and y-components), the time taken to eliminate the target, the finger contact area, and the duration of the touch interaction from the start to the end of the flick. For the drag task, features included tracing accuracy, measured as the average distance between the input coordinates and the nearest point on the reference spiral centerline. Additionally, we derived input velocity and acceleration based on the coordinate movement, along with the finger contact area and the total duration of the drag interaction.

Common to all tasks, we used inertial sensor data, including acceleration, angular velocity, and orientation. Linear interpolation was applied to handle any missing values. To capture the hand movement dynamics surrounding the interaction, sensor data were extracted from 3 specific temporal windows: “preinput” (the 10 frames immediately preceding the input event), “during-input” (the average value recorded at the time of input), and “postinput” (the 10 frames immediately following the event) windows. Subsequently, we performed statistical calculations on these extracted data streams to derive the final feature set. These calculations included the differences between consecutive frames to capture the rate of change, as well as the maximum, minimum, range, and mean values to summarize the signal characteristics over the period of interest.

### Data Processing

The target variable for our predictive models was the grip strength value, derived by averaging the 2 measurements taken within each set. We then constructed 4 distinct regression models by pairing this grip strength data with the extracted behavioral features. First, we developed task-specific models, which were trained exclusively on data from each interaction type—namely, the tap, flick, and drag tasks. These models allowed us to evaluate the predictive power of each gesture independently. Second, we constructed a unified model trained on a combined dataset incorporating features from all 3 tasks. By integrating multimodal features across different interaction styles, this unified approach aimed to enhance overall prediction accuracy and establish a comprehensive model capable of estimating grip strength from a variety of daily smartphone gestures.

In this study, we used all the extracted features (touch interaction features, device motion features, and temporal metrics) for model training without applying dimensionality reduction or exclusion techniques. We relied on the inherent feature importance evaluation capabilities of nonlinear models (eg, random forest) to handle potential multicollinearity.

To remove high-frequency noise from the raw sensor data, we applied a low-pass filter with a cutoff frequency of 20 Hz. Before being used as input for the machine learning models, all extracted features were normalized using StandardScaler (scikit-learn; version 1.2.2) to ensure that each feature contributed equally to the model training. For each machine learning model, we performed a grid search to identify the optimal hyperparameters. This systematic search allowed us to select the best-performing configuration for each algorithm, ensuring the robustness of our grip strength estimation.

### Model Training and Validation Scenarios

To estimate grip strength, we implemented 4 regression models using the scikit-learn library in Python. The random forest model was configured with 200 estimators, a maximum depth of 20, and the square root of features used for splitting, with the random state fixed at 42 for reproducibility. For support vector regression, we used the radial basis function kernel with a regularization parameter C of 100 and a “scale” kernel coefficient. The gradient boosting (GB) model used 100 estimators, a learning rate of 0.1, and a maximum depth of 5. Finally, the k-nearest neighbors (k-NN) model was set with 9 neighbors, using distance-based weighting and the Manhattan distance metric. Model evaluation was performed using 5-fold cross-validation, comparing performance based on the MAPE and the coefficient of determination.

To evaluate the feasibility of a practical deployment scenario, we assessed the model’s performance when trained on a combination of data from other participants supplemented with varying amounts of the target user’s specific data. Specifically, we conducted evaluations using 2 approaches: leave-one-user-out validation, in which 0 days of the target user’s data were included in the training set, and a personalized calibration approach using 1 to 4 days of the target user’s data (few-day calibration). This experimental design simulates a real-world scenario in which a generic model is calibrated with user-specific data collected during the initial period of use to improve estimation accuracy.

### Ethical Considerations

The study protocol was approved by the Research Ethics Committee of Keio University (2025-041). Prior to the experiment, the detailed purpose and procedures of the study were explained to all participants, and written informed consent was obtained. Participants were informed that they could withdraw from the study at any time without penalty. Regarding the exclusion criteria, individuals with preexisting hand impairments or pain were excluded from participation due to safety considerations. As the data collection involved repeated muscle exertion for maximum grip strength measurement, this exclusion was strictly enforced to prevent any risk of injury or symptom exacerbation. All participant data were anonymized and stored on a secure server. Each participant received a cash compensation of ¥2000 (approximately US $13) for their time and participation in the study.

## Results

### Collected Data

In total, 1050 data points (10 repetitions × 5 days × 21 participants) were collected during the measurements for each tap, flick, and drag task. We used grip strength measured before each task as the ground truth, and we mapped this value onto the data collected in each of the 10 executions of the task. Therefore, each data point consisted of a ground-truth grip strength value and data collected through 1 execution of the task. The average time taken for each task was 6.11 (SD 1.37) seconds for the tap task, 7.45 (SD 1.26) seconds for the flick task, and 3.84 (SD 1.88) seconds for the drag task.

### Random Split Cross-Validation Result

**Figure 5:**
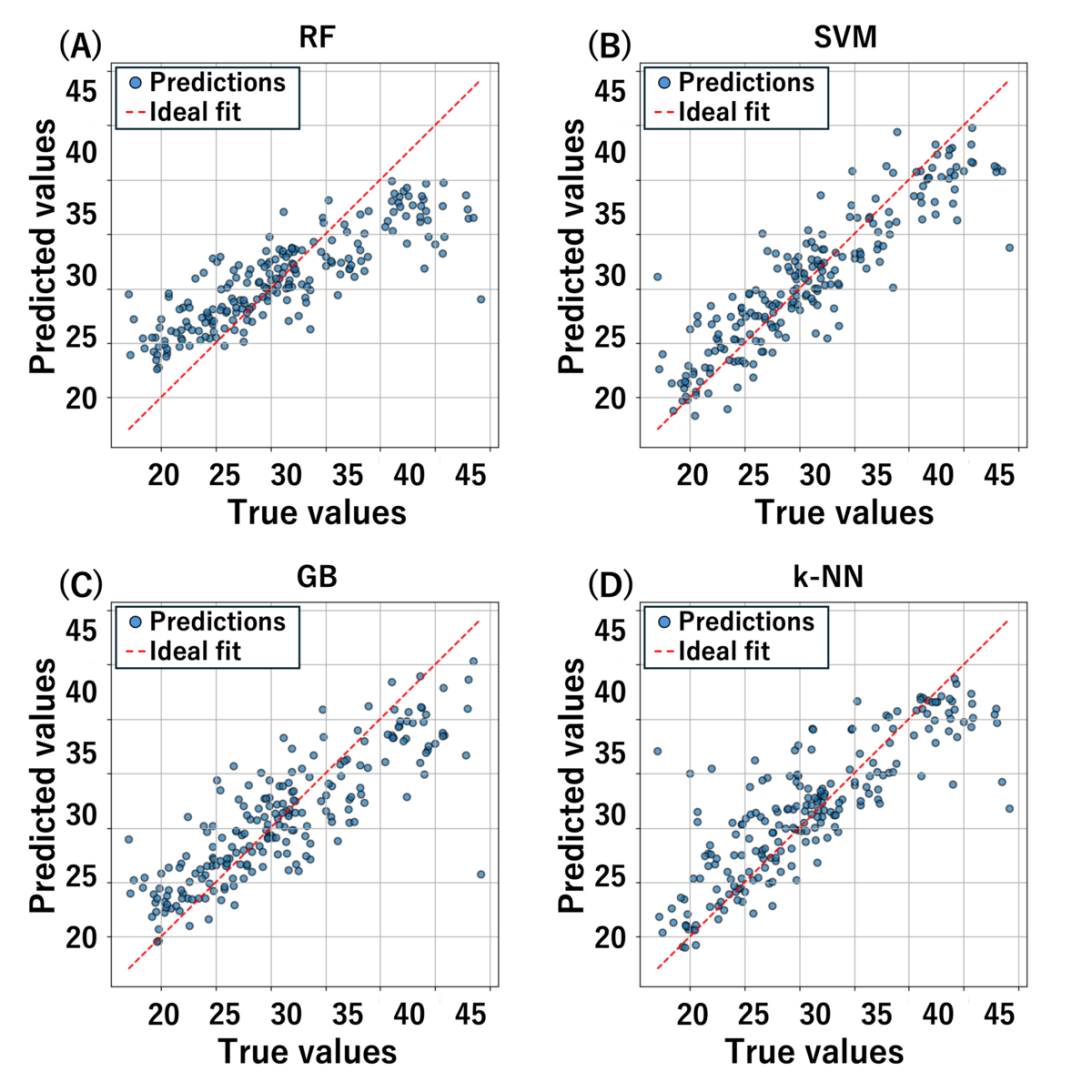
Comparison of true and predicted values of grip strength (kg) in the unified model. GB: gradient boosting; k-NN: k-nearest neighbors; RF: random forest; SVM: support vector machine.

Table 1 shows the MAE, Table 2 shows the MAPE, and Table 3 shows the coefficient of determination. In all tasks, the support vector machine (SVM) achieved the best results in terms of both MAPE and coefficient of determination. Additionally, the unified model, which combined features from different tasks, showed a lower MAPE than single-task models. It also achieved the highest coefficient of determination. Figure 5 illustrates a scatter plot of the true values (x-axis) and the estimated values (y-axis) for the test data of the unified model. SVMs, GB, and k-NNs had an average error of approximately 3 kg. For random forest, there were many data points with prediction errors of ≥5 kg for both low and high true values.

**Table 1:** Mean absolute error of grip strength estimation models across different smartphone tasks.

Task	Random forest (kg), mean absolute error (SD)	Support vector machine (kg), mean absolute error (SD)	Gradient boosting (kg), mean absolute error (SD)	k-nearest neighbors (kg), mean absolute error (SD)
Tap	3.62 (0.24)	3.17 (0.20)	3.27 (0.29)	3.48 (0.29)
Flick	3.99 (0.23)	3.52 (0.17)	3.57 (0.20)	3.60 (0.34)
Drag	3.84 (0.31)	3.46 (0.26)	3.54 (0.31)	3.48 (0.24)
All	3.48 (0.21)	2.62 (0.18)	3.07 (0.28)	2.90 (0.12)

**Table 2:** Mean absolute percentage error for the proposed estimation method by task type.

Task	Random forest (%), mean absolute percentage error (SD)	Support vector machine (%), mean absolute percentage error (SD)	Gradient boosting (%), mean absolute percentage error (SD)	k-nearest neighbors (%), mean absolute percentage error (SD)
Tap	12.03 (0.51)	10.74 (0.65)	10.91 (0.63)	11.64 (1.19))
Flick	13.24 (0.55)	11.75 (0.77)	11.94 (0.61)	12.01 (1.25)
Drag	13.02 (0.87)	11.55 (0.71)	11.88 (0.86))	11.69 (0.77)
All	11.87 (0.36)	8.91 (0.57)	10.47 (0.84)	10.15 (1.25)

**Table 3:** Coefficient of determination (R2) for the performance evaluation of regression models.

Task	Random forest, *R*^2^ (SD)	Support vector machine, *R*^2^ (SD)	Gradient boosting, *R*^2^ (SD)	k-nearest neighbors, *R*^2^ (SD)
Tap	0.610 (0.024)	0.693 (0.039)	0.675 (0.031))	0.650 (0.099)
Flick	0.567 (0.028)	0.644 (0.042)	0.634 (0.030)	0.625 (0.106)
Drag	0.570 (0.033)	0.659 (0.025)	0.648 (0.046)	0.634 (0.012)
All	0.638 (0.026)	0.802 (0.036)	0.731 (0.042)	0.706 (0.095)

### Leave-One-User-Out and Few-Day Calibration Validation Results

**Figure 6:**
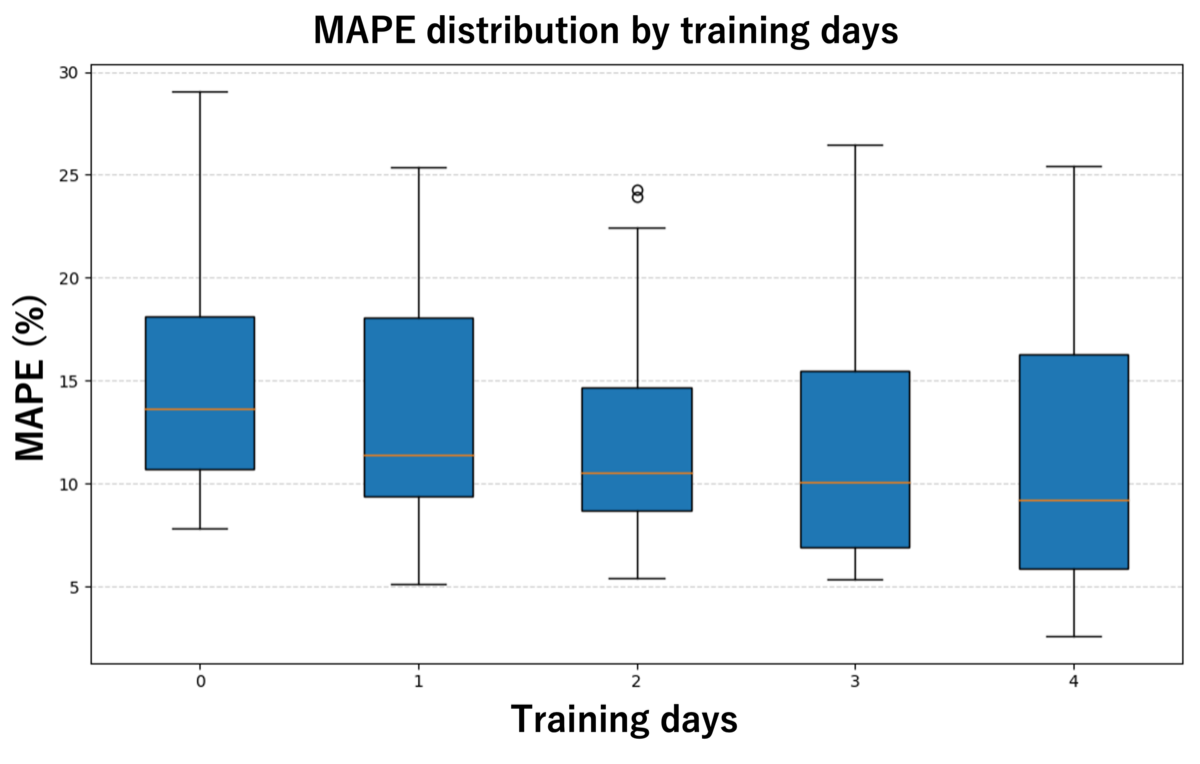
Distribution of estimation errors from the initial state to 4 days of calibration. The box plots illustrate the mean absolute percentage error (MAPE) of the support vector model as the number of user-specific training days increases from 0 to 4. The case with 0 days represents leave-one-user-out validation, while cases with 1 to 4 days represent few-day calibration validation. The horizontal line within each box represents the median error, and the box edges represent the IQR.

For the leave-one-user-out and few-day calibration validations, the SVM model was used and evaluated based on the MAE and MAPE for each participant. As shown in Table 4 and Figure 6, the estimation accuracy consistently improved as more days of the target user’s data were incorporated into the training set. In this evaluation, the case with 0 training days corresponds to leave-one-user-out validation, while the cases with 1 to 4 training days represent the few-day calibration validation.

**Table 4:** Comparison of mean absolute error and mean absolute percentage error by number of training days.

Variable	Day 0	Day 1	Day 2	Day 3	Day 4
Mean absolute error (kg; SD)	5.03 (2.65)	4.39 (2.28)	3.96 (2.11)	3.80 (2.04)	3.56 (2.23)
Mean absolute percentage error (%; SD)	15.08 (5.40)	13.96 (5.57)	12.60 (5.54)	12.23 (5.68)	11.64 (5.80)

### Subjective Workload Assessment

To evaluate the subjective workload of the measurement tasks using our proposed method, we administered the National Aeronautics and Space Administration Task Load Index (NASA-TLX) questionnaire to 9 participants. Prior to the assessment, participants were instructed on how to use the app and performed the tap, flick, and drag tasks once. Subsequently, they completed the questionnaire. Following the raw Task Load Index method, participants rated 6 subscales on an 11-point scale ranging from 0 (low) to 10 (high). The results are summarized in Table 5. The overall workload score (mean of all subscales) was 3.04 (SD 2.23), indicating a low level of workload overall. Among the subscales, mental demand (mean 1.89, SD 2.02), effort (mean 1.78, SD 2.10), and frustration (mean 1.78, SD 2.10) were rated particularly low. In contrast, temporal demand showed the highest mean value at 5.11 (SD 3.14).

**Table 5:** National Aeronautics and Space Administration Task Load Index scores.

Subscales	Descriptions	Mean (SD)
Mental demand	How much mental and perceptual effort did the task require?	1.89 (2.02)
Physical demand	How much physical activity was required?	4.22 (2.86)
Temporal demand	How much time pressure did you experience while performing the task?	5.11 (3.14)
Performance	How successful were you in accomplishing the task?	3.44 (2.50)
Effort	How hard did you have to work?	1.78 (2.10)
Frustration	How irritated, stressed, and annoyed did you feel?	1.78 (2.10)
Overall workload	Average of the 6 subscales	3.04 (2.23)

## Discussion

### Model Performance and Estimation Characteristics

In the model comparison, SVMs achieved the highest accuracy, followed by GB, k-NNs, and random forest. SVMs and GB effectively captured nonlinear relationships in the data, enabling them to achieve high estimation performance despite the limited dataset. In contrast, k-NNs tend to suffer from decreased accuracy in distance calculations when applied to high-dimensional data, leading to lower overall precision. Additionally, random forest does not focus on reducing errors in the same way as boosting methods, which may result in suboptimal feature selection.

Furthermore, there was a tendency for grip strength to be overestimated when the true value was low and underestimated when the true value was high. This may be attributed to distributional bias in the original data. Most of the data were concentrated between 25 kg and 35 kg, while values >40 kg or <20 kg were relatively scarce. Consequently, the model was likely influenced toward the mean, reducing the estimation accuracy for extreme values.

The observed reduction in estimation errors as the number of calibration days increased suggests that user-specific data significantly enhance the model’s ability to account for individual variability. Compared to the leave-one-user-out validation, which yielded the highest error rate, the introduction of even a single day of user data led to a notable improvement. The most substantial improvement occurred between the initial state and the first 2 days of calibration, after which the error rate continued to decrease more gradually. This indicates that while initial personalization is critical to overcoming the limitations of a purely generic model, the system reaches a state of relative stability with approximately 3 to 4 days of data.

The primary dataset involved university students aged 20 to 24 years. To assess the applicability of our framework to broader demographics, we conducted a preliminary case study with a 53-year-old participant (middle-aged adult). We trained a model using the participants’ data from 1 day, combined with the entire dataset from the younger group, and tested it on the participants’ data from other days. The model achieved an estimation error with an MAE of 1.87 kg (MAPE 11.19%), which is comparable to the accuracy levels obtained from the younger participants. Regarding participants with hand disabilities, we were unable to include them in this study due to ethical considerations. As the data collection tasks involve repeated muscle exertion and impose a certain physical load on the hand, recruiting individuals with existing hand impairments posed a potential risk of exacerbating their conditions.

### Practical Applications and Future Directions

In the future, we will implement the proposed model on a smartphone and evaluate its practicality for daily grip strength monitoring. Specifically, we plan to develop a system that inputs data obtained after certain operations into the model and provides feedback on the estimated results. The goal is to track changes in grip strength and use the data for health management and exercise. To achieve this, maintaining accuracy on actual devices and assessing usability will be crucial challenges.

For daily grip strength monitoring, we believe that ease of measurement is one of the most crucial factors. When using the unified model (ie, conducting all tasks), the required time is approximately the same as that for completing one set of smartphone tasks in the data collection experiment. The average completion time for these tasks in our experiment was approximately 30 seconds, which we consider short enough to avoid burdening users in everyday use.

Furthermore, numerous studies have reported on the relationship between smartphone use and grip strength variations, revealing that individuals who spend more time using smartphones daily tend to have lower grip strength [14-16]. Excessive smartphone use can affect hand function and may lead to conditions such as tenosynovitis [17,18]. By using this system, it may be possible to detect grip strength decline during smartphone use, estimate hand fatigue, encourage appropriate use, and reduce the risk of orthopedic disorders.

### Subjective Workload and Usability

To evaluate user fatigue and task burden during daily monitoring, we conducted a subjective workload evaluation using the NASA-TLX with 9 participants. The overall workload score was low at 3.04 (SD 2.23) (on a scale of 0 to 10). Specifically, the physical demand score averaged 4.22 (SD 2.86). Given that the task inherently involves physical muscle exertion to estimate grip strength, a moderate level of physical load is expected. However, this value indicates that the task does not impose excessive fatigue compared with maximum voluntary contraction tasks typically performed with a conventional dynamometer.

Notably, the mean temporal demand score was relatively high at 5.11 (SD 3.14), even though participants were explicitly instructed to perform the tasks at their own self-selected pace. This perception may be attributed to the app’s feedback design, specifically the display of elapsed time upon task completion. Although participants were instructed to prioritize their own pace, the visualization of a time metric likely created an awareness of *being timed*, inducing a psychological pressure to perform quickly. Consequently, to mitigate this unintended pressure, we plan to redesign the user interface by removing the explicit display of elapsed time in future versions of the app.

Crucially, however, the frustration score remained extremely low at 1.78 (SD 2.10). The clear dissociation between high temporal demand and low frustration implies that this time pressure was not perceived as negative stress or panic but potentially as a gamified element or a source of positive engagement to complete the task efficiently. These results confirm that our proposed method is suitable for continuous daily use without causing significant user burden or stress.

### Limitations

To evaluate the generalization of the proposed model across different participants, leave-one-user-out validation was performed. As reported in the Results section, this approach yielded a relatively large estimation error, with an MAE of 5.03 (SD 2.65) kg and an MAPE of 15.08% (SD 5.40%) for the SVM. These results, particularly when contrasted with the improved accuracy observed after just 1 to 4 days of calibration, highlight the significant impact of individual variability in grip strength and smartphone interaction patterns. The high error rate in leave-one-user-out validation suggests that a purely generic model struggles to adequately capture the unique features of each individual’s operations. Consequently, our findings indicate that while the model provides a strong foundation, an individual calibration process is currently essential to bridge the gap between a general estimation and the high precision required for practical, personalized health care monitoring.

The accuracy of the proposed model may depend on the smartphone model used. The extracted features, such as device acceleration and angular velocity, could vary due to differences in device size, weight, and screen dimensions. Consequently, the model may not achieve the same level of accuracy when applied to a different smartphone model. Additionally, the participants in this study used different smartphone models in their daily lives. Some participants struggled with input due to differences in size and shape compared with their usual devices. Their unfamiliarity with the device may have affected task performance. Therefore, it is necessary to explore methods to mitigate the impact of device-related constraints on model performance.

In our experiment, participants were required to hold the smartphone with 1 hand and use their thumb for input. This setup was chosen considering the ease of one-handed operation and the need for consistency in analysis. Meanwhile, factors such as finger length and hand size were not examined in this study. However, because these elements are known to influence touch input accuracy [19], factoring in such variations may improve the accuracy of grip strength estimation across diverse users.

Additionally, using 1 hand with the thumb was the primary interaction style in our experiment. Consequently, the current model is optimized exclusively for this specific posture. We recognize that users often use alternative styles, such as holding the device with 1 hand and operating it with the index finger of the other, which can significantly alter sensor data profiles. To ensure data consistency in a practical deployment, we propose implementing on-screen instructions or a brief tutorial to explicitly guide users to adopt the required one-handed thumb posture during the measurement tasks. Expanding the model to accommodate diverse interaction styles remains an important direction for future work.

In this study, data collection was conducted exclusively with right-handed participants operating the smartphone with their dominant (right) hand. To extend the applicability of our model to left-handed users operating with their dominant hand, we propose a geometric transformation approach. As the anatomical structure of the hands is symmetrical, left-hand interaction data can be mapped to the right-hand feature space by mirroring the horizontal coordinates (x-axis) of touch points and reversing the rotation direction of the spiral drag task (ie, treating counterclockwise input as clockwise). This preprocessing allows the model trained on right-dominant data to be directly applied to left-dominant users.

However, the validity of estimating grip strength using the nondominant hand (regardless of whether it is the left or right hand) remains unverified, as differences in dexterity and motor control may significantly alter sensor patterns compared with the dominant hand.

Consequently, to ensure valid estimation in a real-world deployment, the app requires an input interface where the user explicitly specifies two parameters before use: (1) which hand is currently being used (left or right) and (2) whether it is their dominant or nondominant hand. On the basis of these inputs, the system can determine whether to apply geometric transformations (for left-dominant input) or to notify the user that the current condition (nondominant input) is outside the verified scope of the model.

We acknowledge that this study used a single smartphone model (Pixel 7a). As inertial sensor data (acceleration and angular velocity) are physically influenced by the device’s weight and dimensions, verifying reproducibility on different devices is crucial. However, the Pixel 7a (weight 193.5 g; display 15.5 cm) represents a standard form factor in the current smartphone market. Therefore, we anticipate that the fundamental physical interactions between grip strength and device motion would remain consistent, and the influence on estimation accuracy would be limited for devices with similar specifications.

To strictly address the issue of varying sensor characteristics (eg, bias or sensitivity) across different hardware in the future, we propose 3 potential approaches. First, sensor calibration could be introduced as a preprocessing step to identify and correct for device-specific sensor characteristics, thereby standardizing the input data across different models. Second, we suggest focusing on robust feature extraction, which prioritizes features that are independent of raw sensor hardware variations, such as relative motion patterns, rather than relying on absolute sensor values. Finally, if a large-scale dataset across multiple devices becomes available, device-specific features—such as model ID, weight, or screen size—could be explicitly incorporated as input variables to train a more generalized and hardware-agnostic model.

### Comparison With Prior Work

Several studies have explored methods for estimating grip strength without using a grip dynamometer. Matsumoto et al [20] proposed a method for estimating grip strength by capturing an image of a hand gripping a soft tennis ball using a monocular camera on a smartphone and analyzing changes in the angle of finger joints. Yamamoto et al [21] proposed a grip strength measurement method using capacitive touch sensors on smartphones, which does not require any electronic components on the measuring device itself. Although these methods offer alternative approaches to measuring grip strength, they still require external equipment beyond a smartphone, and the measurement results may be influenced by the user’s posture. In this study, our objective is to estimate the grip strength using only the smartphone, with the aim of establishing an easy and convenient measurement method without specialized equipment.

This study examines the estimation errors of the proposed grip strength model. The Jamar dynamometer manufacturer recommends a measurement error within 5% [22], whereas the error rate in this study slightly exceeds this threshold. We also evaluated the clinical relevance of our estimation accuracy. The minimal clinically important difference for grip strength is reported to be between 5.0 kg and 6.5 kg [23]. Our model achieved an MAE of 2.62 (SD 0.18) kg in the random split and 4.39 (SD 2.28) kg in the single-day case of the few-day calibration, both falling within this minimal clinically important difference range. This indicates that our method is effective for detecting meaningful changes in muscle strength over time, such as recovery or decline. However, a critical limitation arises when applying this method to disease screening based on specific thresholds. For instance, the Asian Working Group for Sarcopenia 2019 defines diagnostic cutoffs of <28 kg for male and <18 kg for female individuals [24]. Given our estimation error of approximately 2.6 kg to 4.4 kg, there is a risk of misclassification (false positives or false negatives) for individuals whose grip strength lies near these borderline values. Consequently, we position our method not as a replacement for clinical-grade dynamometers for definitive diagnosis but rather as a pervasive tool for daily longitudinal monitoring and initial screening to identify at-risk individuals who should seek professional assessment.

The accuracy of the proposed model is compared with previous grip strength estimation studies. Park et al [25] used national survey data from >20,000 participants, where grip strength was measured using a digital grip strength dynamometer. Their analysis used age, sex, hand preference, height, weight, waist circumference, and 5 physical activity levels (vigorous or moderate work, vigorous or moderate recreation, and travel) as input features, achieving a coefficient of determination of 0.717 with Extreme Gradient Boosting. Hwang et al [26] developed a deep learning model trained on data from 164 participants, incorporating various postures, demographic information, and anthropometric data as input, achieving a coefficient of determination of 0.88. This study, in contrast, focuses on estimating grip strength using everyday smartphone interactions, making the input data fundamentally different from those used in previous studies. However, the limited number of participants and the small training dataset present challenges in terms of reliability and accuracy. Additionally, further investigation into user-independent models is necessary. Future work should aim to expand the dataset and improve the model to enhance estimation accuracy.

### Conclusions

In this study, we proposed a model for estimating grip strength using smartphone input data. First, grip strength was measured, followed by the execution of smartphone tasks, during which sensor data from the device were collected. On the basis of the obtained data, multiple machine learning models were constructed and evaluated. The results showed that the model using SVMs achieved the highest accuracy. Furthermore, integrating features obtained from different tasks improved estimation accuracy. In the future, we aim to implement the model on a smartphone to enable real-time grip strength estimation. To achieve this, further accuracy improvements and evaluations under various conditions will be necessary.

## Data Availability

The source code for the smartphone app developed in this study and the data analysis scripts are available in the GitHub repository [27].

## Appendix

Multimedia Appendix 1 Video demonstrating the smartphone tasks (tap, flick, and drag) and providing an overview of the grip strength estimation method.

## Abbreviations

GB: gradient boosting
k-NN: k-nearest neighbors
MAE: mean absolute error
MAPE: mean absolute percentage error
NASA-TLX: National Aeronautics and Space Administration Task Load Index
SVM: support vector machine
